# Sustained Glycemic Control With Ivacaftor in Cystic Fibrosis–Related Diabetes

**DOI:** 10.1177/2324709619842898

**Published:** 2019-04-22

**Authors:** Francis Christian, Andrew Thierman, Erin Shirley, Karen Allen, Cory Cross, Kellie Jones

**Affiliations:** 1 University of Oklahoma Health Sciences Center, Oklahoma City, OK, USA; 2 University of Oklahoma, Oklahoma City, OK, USA

**Keywords:** cystic fibrosis–related diabetes, ivacaftor, cystic fibrosis

## Abstract

Cystic fibrosis–related diabetes (CFRD) is a common comorbidity in cystic fibrosis with pancreatic insufficiency occurring early in the disease process. Current treatment is exogenous insulin therapy as CFRD is due to impaired insulin secretion. Recent small studies have shown improvement in endogenous insulin secretion with a short period of ivacaftor therapy in primarily pediatric patients with cystic fibrosis transmembrane conductance regulator mutations amenable to potentiation. In this article, we present the case of an adult patient with long-standing CFRD who developed sustained improvement in glycemic control after initiation of ivacaftor.

## Introduction

In cystic fibrosis (CF), patients have thick, viscous secretions, causing fibrosis and scarring. Such exocrine pancreatic damage leads to reduced β-cell levels and insulin deficiency,^1^ resulting in CF-related diabetes (CFRD). Peripheral sensitivity to insulin is less extensively affected as insulin production. As pulmonary function declines in CF, insulin resistance becomes more severe.^2^ Stable CF outpatients are evaluated for CFRD started age 10 with primary recommendation for 2-hour oral glucose tolerance test (OGTT).^3^ The recommended treatment for CFRD is insulin therapy, similar to type 1 diabetes mellitus, since oral hypoglycemic medications have been found to be ineffective. Treatment goals have been set at maintaining a hemoglobin A1c (HbA1c) level less than 7%.^3^ Though insulin therapy has been proven effective at controlling blood sugar levels, it is not a cure-all. Patients with CFRD do develop microvascular complications, albeit with a slightly lower prevalence than those with other forms of diabetes.^4^

Ivacaftor is a therapeutic agent for patients with specific mutations in the CF transmembrane receptor (CFTR) protein. Patients with class III mutations (defective regulation/gating of the channel) are the most susceptible, as ivacaftor increases ion flow through the protein channel. Ivacaftor has been proven to positively affect lung and exocrine pancreas function in CF patients^5^ and through unclear mechanisms may improve endocrine function.

## Case

A 34-year-old male diagnosed with CF as a child was found to have CFRD at age 20 after joining our tertiary care clinic. He was diagnosed with CFRD based on fasting glucose and HbA1c levels along with symptoms of polyuria and polydipsia. He was started on insulin therapy the year following diagnosis (Table 1) with 1 unit of rapid acting insulin analogue, insulin aspart, per 20 g of carbohydrates, and no basal insulin. Eight years after being diagnosed with CFRD, he was approved to start a new therapy, ivacaftor 150 mg orally twice daily for treatment of his CF based on his G551D mutation. Pre-ivacaftor, his insulin regimen was unchanged as he generally averaged between 4 and 6 units of insulin aspart per meal consistent with a carbohydrate content of 100 to 120 g per meal. This dose was consistent with what he received as an inpatient during admissions with stable postprandial levels not requiring additional correction. Within 6 months of starting ivacaftor, he reported recurrent hypoglycemic episodes and stopped insulin therapy. Between starting ivacaftor and the 3 subsequent years, the patient had been hospitalized for CF exacerbations 8 times at our institution. On these admissions, he rarely required insulin with only low-dose sliding scale insulin aspart as needed for elevated blood sugars. Fasting blood sugars during these exacerbations on ivacaftor were variable but similar to those pre-ivacaftor, with fasting blood sugars ranging between 70 mg/dL and 140 mg/dL. His HbA1c levels were monitored at each of these admissions (Table 2). During 2 of these exacerbations, he did receive single-dose intravenous methylprednisolone in the emergency room prior to admission: June 2012 and May 2015. On all other exacerbations, he was admitted directly from clinic for intravenous antibiotics without steroid administration. Notably, he did have sinus infection in 2015 and received PO (per os) dexamethasone from otolaryngology service. Due to concern for medication-associated hypoglycemia, fluoroquinolones and sulfamethoxazole and trimethoprim were avoided when possible. In the first 4 years after receiving ivacaftor, he did not receive sulfamethoxazole and trimethoprim. He did eventually receive a course of sulfamethoxazole and trimethoprim as an outpatient in June 2017 as part of therapy for sinus-related issues. He was lost to follow-up for approximately 11 months during his fourth year of therapy with ivacaftor. On reestablishing care, he had a random finger-stick blood glucose >200 mg/dL with HbA1c 6.5% and was restarted on insulin aspart, 1 unit per 25 g of carbohydrates. At subsequent follow-up appointments, he complained of struggling with highly variable self-monitoring blood glucose levels ranging from 70 to 300 mg/dL, with the lows occurring postprandially and with exertion. His fasting finger-stick blood glucose values were consistently around 100 mg/dL. He was advised to follow a low-carbohydrate diet with <20% calories from carbohydrates as it was thought increased simple carbohydrates was increasing glucose and insulin secretion postprandially. On returning to diabetic specialty clinic over the past year, he had elevated HbA1c measurements of 8.8% and 8.6%, respectively. His current regimen consists of long-acting insulin, glargine 5 units at night, and rapid acting insulin aspart, 6 units before meals. Prior to starting ivacaftor, the patient had a consistent decline in lung function (Table 1), which improved after starting therapy (Table 2). Concurrent weights are provided in Tables 1 and 2 showing consistently higher weights during ivacaftor therapy.

**Table 1.** Pre-Ivacaftor HbA1c levels.

Date	Pre-Ivacaftor HbA1c^a^	Weight (kg)	Post BD FEV1 (%)
May 2005	5.9%	63	70
May 2006	6.0%	67	69
February 2007	5.8%	65	63
June 2007	6.0%	70	69
March 2008	5.9%	70	50
August 2009	6.4%	68	53
May 2010	5.9%	76	51
October 2010	6.6%	72	55
March 2011	6.2%	68	48
September 2011	6.4%	63	38

Abbreviations: HbA1c, hemoglobin A1c; BD, bronchodilator; FEV1, forced expiratory volume in 1 second.

a All measurements were taken with patient on insulin.

**Table 2.** Post-Ivacaftor HbA1c levels.

Date	Post-Ivacaftor HbA1c	Weight (kg)	Post BD FEV1
June 2012^a^	6.0%	73	43%
January 2013^a^	6.1%	78	61%
April 2013^a^	5.6%	78	58%
June 2014^a^	5.6%	80	57%
May 2015^a^	6.5%	81	56%
April 2016^b^	6.5%	78	64%
October 2016^b^	6.0%	78	55%
May 2017^b^	6.6%	80	55%

Abbreviations: HbA1c, hemoglobin A1c; BD, bronchodilator; FEV1, forced expiratory volume in 1 second.

a Values are without insulin treatment.

b Values are with insulin treatment.

## Discussion

Improvement in CFRD with ivacaftor therapy has been shown in small studies in young patients where insulin secretion may be impaired but not extremely defective.^6^ Our report describes a patient diagnosed with CFRD who found sustained glycemic control off insulin for a period of 3 years, while on ivacaftor therapy. The exact pathophysiologic mechanism of CFRD has never been quite elucidated. It has been postulated that with exocrine pancreatic damage, there is a loss of pancreatic β-cells.^7^ However, the surviving β-cells should provide sufficient function for euglycemia. If there is in fact a functional deficiency in the surviving β-cells, then functional restoration may result in restored insulin secretion and euglycemia.

A recent comprehensive study has shown that pancreatic β-cells do not directly express the CFTR.^8^ Therefore, it does not follow that ivacaftor improves insulin secretion directly by increasing CFTR expression. Even in a severely damaged pancreas due to secretions and fibrosis, with significant survival of functioning β-cells, the environment they exist in is sufficiently abnormal to prevent their proper functioning.^9^ The combination of lack of CFTR channels and the abnormal environment point toward an indirect mechanism for ivacaftor’s improvement in insulin secretion. Possible indirect mechanisms include relief of islet inflammation, improved secretion of incretins, glucagon-like peptide-1 (GLP-1) and glucose-dependent insulinotropic polypeptide (GIP), and a return to a more normal pancreatic environment. A study done measuring incretin levels with and without ivacaftor showed increased levels of GLP-1 and GIP but could not definitively prove causation.^8^ Given that ivacaftor therapy can positively affect all 3 mechanisms, it is possible that slight improvements in each is responsible for the significantly improved control of insulin release and glycemia.

Screening for CFRD should begin at 10 years of age, and it should occur annually using a 2-hour 75 g OGTT for screening as opposed to a HbA1c measurement.^3^ Screening with OGTT is for 2 major reasons. The first reason is primary morbidity from CFRD stems from nutritional and pulmonary effects of diabetes,^10^ rather than microvascular or macrovascular complications. The body’s response to elevated insulin as a consequence of glucose loads is of more importance than basal insulin production. Second, multiple studies have shown that HbA1c levels do not always correlate with CFRD severity.^11^

According to recent guidelines, diagnosing CFRD in stable outpatients with CF follows standard American Diabetic Association criteria; the same as in patients without CF. Patients found to have a fasting plasma glucose ≥126 mg/dL, a 2-hour OGTT ≥ 200 mg/dL, or a HbA1c ≥ 6.5% on 2 occasions meet the criteria.^3^ A random plasma glucose level ≥200 mg/dL along with symptoms of hyperglycemia (polydipsia and polyuria) can also be used.

Our patient was restarted on insulin therapy based on an elevated HbA1c level and a random blood glucose level >200 mg/dL. The patient’s HbA1c levels were similar before and after starting ivacaftor. However, he reported multiple episodes of hypoglycemia after starting ivacaftor, prompting cessation of insulin therapy. The episodes of hypoglycemia were almost always after meals, and rarely during periods of fasting, indicating ivacaftor’s effect on insulin secretion is greatest on insulin release in response to a glucose load. It also implies CF patients taking targeted CFTR potentiating medications may have adequate control of their postprandial insulin levels without requiring exogenous insulin.

Our patient with diagnosed CFRD maintained a HbA1c level within treatment range for more than 3 years without insulin treatment. Despite the fact that pulmonary function and insulin resistance have been found to trend together,^12^ he never developed any evidence of microvascular complications, even though he was hospitalized with CF exacerbations at least once every year.

Ivacaftor has been proven to improve exocrine pancreas function,^13^ and dysfunctional exocrine pancreas function is the major driving force behind endocrine pancreas dysfunction seen in CF patients. In the case presented, ivacaftor maintained glycemic control in CFRD, by enhancing endogenous insulin release and mitigating the need for exogenous insulin. This case highlights the need for patients on ivacaftor to be monitored extremely closely while concurrently on insulin as they may be at increased risk of hypoglycemia.

Ivacaftor is not effective in treating all forms of CF. It is most specifically used to treat patients with the G551D mutations,^5^ resulting in failure of the CFTR to open at the cell surface. G551D mutations have a prevalence of approximately 4% to 5% in the CF population.^14^ Presently, ivacaftor has not been shown to be effective in patients with other types of CFTR mutations. Most recently, the combination therapy lumacaftor/ivacaftor has been approved for treatment on CF patients with delta 508 mutations, which comprise approximately 70% of all mutations.^15^ If in addition to improved lung function, sustained glycemic control is seen in patients receiving lumacaftor/ivacaftor, CF patients may have preserved endocrine and exocrine pancreatic function and may be less likely to develop CFRD.

## Conclusion

This case demonstrates that in patients with CFRD and mutations amenable to CFTR modulating therapy, management may not require the use of exogenous insulin. Ivacaftor, which improves lung and pancreas function in CF patients with class III mutations, may serve to maintain euglycemia by enhancing endogenous insulin production. In patients who are already on insulin therapy for CFRD, practitioners should remain mindful of the potential for hypoglycemia with ivacaftor.
